# Radioimmunotherapy: a game-changer for advanced non-small cell lung cancer

**DOI:** 10.3389/fimmu.2024.1522508

**Published:** 2024-12-06

**Authors:** Huichan Xue, Yunshang Chen, Yun Zhou

**Affiliations:** ^1^ Cancer Center, Union Hospital, Tongji Medical College, Huazhong University of Science and Technology, Wuhan, China; ^2^ Institute of Radiation Oncology, Union Hospital, Tongji Medical College, Huazhong University of Science and Technology, Wuhan, China; ^3^ Hubei Key Laboratory of Precision Radiation Oncology, Wuhan, China; ^4^ Department of Pediatric Surgery, Union Hospital, Tongji Medical College, Huazhong University of Science and Technology, Wuhan, China

**Keywords:** NSCLC, immunotherapy, radiotherapy, combination therapy, clinical perspective

## Abstract

Lung cancer, particularly non-small cell lung cancer (NSCLC), remains a leading cause of cancer-related deaths, with conventional treatments offering limited effectiveness in advanced stages, due to distant metastases and treatment resistance. Recent advancements in immunotherapy, specifically immune checkpoint inhibitors (ICIs), have shown promise, but their efficacy as standalone therapies are often insufficient. This has led to increased interest in combining ICIs with radiotherapy, known as radioimmunotherapy (iRT), to enhance treatment outcomes. This review explores the mechanisms that underlie the synergy between radiotherapy and immunotherapy. Radiotherapy can induce the “abscopal effect”, eliciting systemic immune responses that reduce tumor burdens outside the treated area. It also increases the expression of major histocompatibility complex class I (MHC-I) on tumor cells, improving immune recognition. Furthermore, radiotherapy can modify the tumor microenvironment by inducing metabolic reprogramming to bolster anti-tumor immunity. We discuss strategies for optimizing iRT, including considerations of radiation doses, fractionation schedules, and treatment site selection, which significantly influence immune responses by enhancing MHC-I expression or promoting T-cell infiltration. Clinical evidence supports the efficacy of iRT in NSCLC and other cancers, though challenges in standardizing treatment protocols and managing side effects persist. Overall, radioimmunotherapy presents a promising approach to improving NSCLC treatment outcomes. Ongoing research into its mechanisms and the refinement of treatment may reshape clinical practice, offering more effective and personalized options for patients with advanced lung cancer. Further studies are essential to validate these findings and optimize therapeutic protocols.

## Introduction

Lung cancer is one of the most common and deadliest malignant tumors worldwide, with an incidence and mortality rate of 11% and 20% in the United States in 2024, respectively, and this disease shows an increasing trend in incidence among females in China (1, 2). Pathologically, it can be broadly categorized into small cell lung cancer (SCLC) and non-small cell lung cancer (NSCLC), with NSCLC accounting for approximately 85% (3). NSCLC includes histological types such as adenocarcinoma and squamous cell carcinoma, with adenocarcinoma being the most prevalent histological subtype (4). In recent years, cancer has become a leading cause of death in China, and the 5-year survival rate for advanced NSCLC remains low (5, 6). Due to the high incidence and low survival rates of NSCLC in China, significant advancements have been made in the treatment of NSCLC in recent years. Surgery remains the primary treatment modality for early to mid-stage NSCLC, while chemotherapy and radiation therapy are recommended as conventional treatments for locally advanced or metastatic NSCLC. The development of targeted therapies has shifted treatment strategies from traditional chemotherapy and radiation towards gene mutation-directed targeted therapies. For example, NSCLC with EGFR gene-sensitive mutations can be treated with tyrosine kinase inhibitors (TKIs) (7, 8). Tumor therapies are evolving rapidly, and combination therapy strategies are becoming an emerging trend (9). In recent years, with the breakthrough progress in immunotherapy, it has also been gradually employed in the treatment of lung cancer patients, notably through the use of immune checkpoint inhibitors (ICIs). However, studies have shown that the efficacy of using ICIs alone may not be satisfactory (10, 11). The response rate of ICI monotherapy in solid tumors is only about 20% due to the development of primary or secondary resistance, the specific mechanisms of which involve the loss of antigens from tumor cells, the infiltration of immunosuppressive cells and the formation of an immunosuppressive microenvironment. Consequently, various methods have been explored to enhance the effectiveness of immunotherapy. In 2015, a study of 41 patients with metastatic solid tumors showed that local radiotherapy combined with granulocyte-macrophage colony-stimulating factor (GMCSF) produced an abscopal effect in 27% of patients, which demonstrates the synergistic effect of immunotherapy and radiotherapy (12). Since then, there has been a growing interest in ICIs combined with radiation therapy (RT), known as radioimmunotherapy (iRT), and many clinical trials have achieved encouraging conclusions (13–15).

## The mechanism of radiotherapy combined with immunotherapy

**Figure 1:**
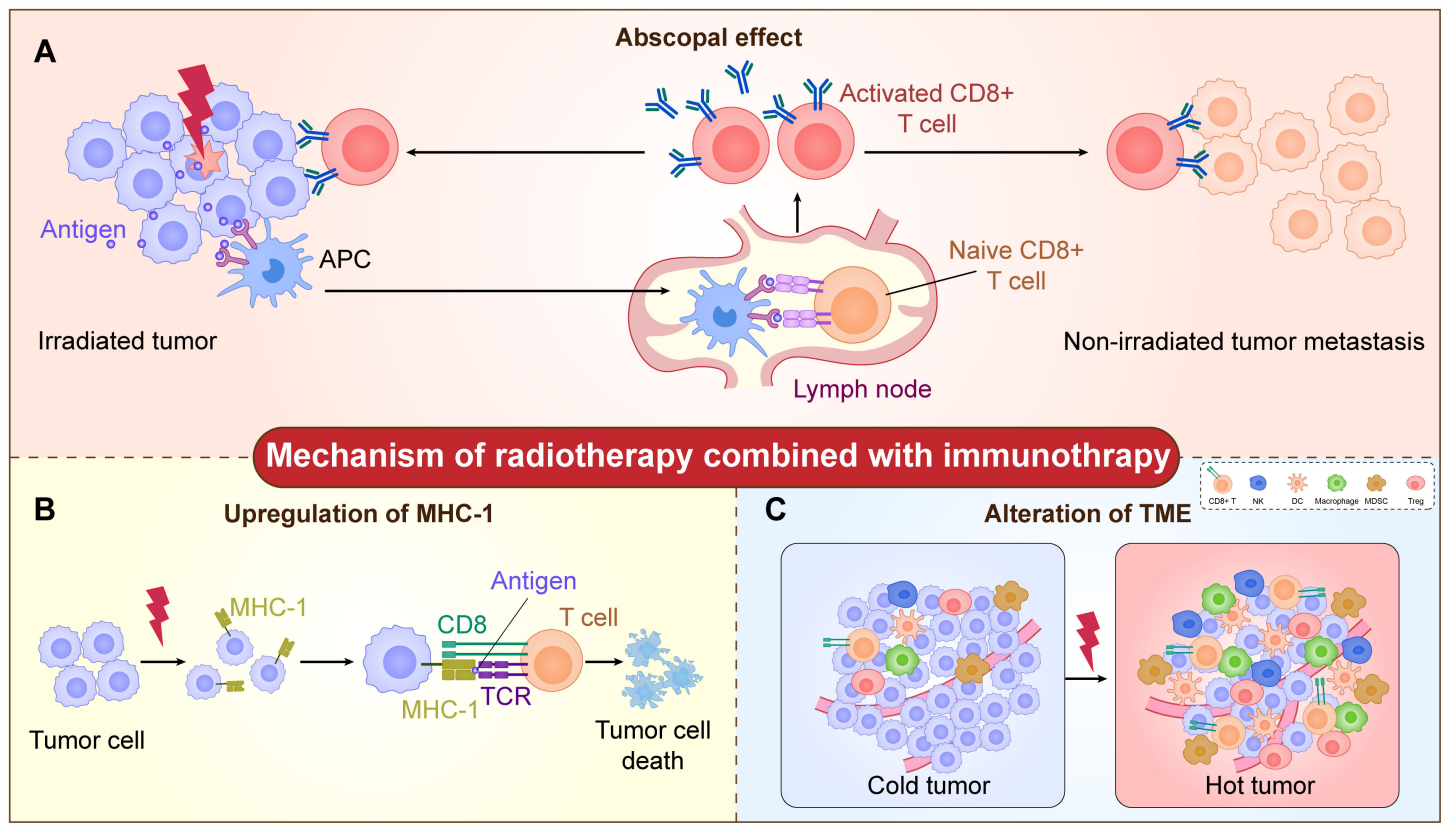
The mechanism of radiotherapy combined with immunotherapy. **(A)** The radiation increases tumor antigen exposure. Antigens from damaged tumor cells can be taken up by antigen-presenting cells (APCs) and presented to native T cells, which leads to the activation of T cells. Subsequently, the activated T cells targeting tumor-specific antigens infiltrate into both the primary tumor and unirradiated metastatic lesions, thereby triggering the abscopal effect. **(B)** The radiation increases the expression of MHC-I on the tumor cell surface and modulates the process of antigen presentation. The MHC-I-positive tumor cells are then recognized and cleared by CD8^+^ T cells. **(C)** The radiation increases the infiltration of immune cells, including CD8^+^ T cells, NK cells, DC cells, macrophages, Treg-cells, MDSCs, et al., which changes the microenvironment from “cold tumor” into “hot tumor”.

Radiotherapy remains one of the essential treatment modalities for malignant tumors (16). “Abscopal effect (AE)” refers to the phenomenon that when a tumor receives local radiotherapy, the distal unirradiated tumor also shrinks or disappears. This observation is attributed to the systemic immune response triggered by local radiotherapy (17, 18). This potential systemic anti-tumor response provides robust evidence for the combination of radiotherapy and immunotherapy. To fully harness the clinical potential of the anti-tumor immune response induced by radiotherapy, it is crucial to understand the mechanism of action of radioimmunotherapy (iRT) (**Figure 1**).

### The “abscopal effect” of radiotherapy

The first clinical case of the AE was reported by Nobler in 1963. A patient with malignant lymphoma received radiotherapy to the abdomen, and the distant bilateral pleural effusions subsided (19). Later, the AE was observed in a series of tumors including non-small cell lung cancer, breast cancer, thymic cancer and melanoma. For example, a lung adenocarcinoma patient with brain metastases underwent radiotherapy to the metastatic lesion. The size of the primary lung lesion was reduced by 40% after completing whole‐brain irradiation (20). The mechanisms of the AE involve complex immune system activation, alterations in the tumor microenvironment, and the interaction of multiple molecular and cellular signals. Radiotherapy-induced immunogenic cell death (ICD) of tumor cells is one of the core mechanisms of AE. ICD refers to the specific way in which tumor cells die in response to injuries such as radiotherapy, releasing a series of damage-associated molecular patterns (DAMPs). These DAMPs have potent immunostimulatory functions and can activate both innate and adaptive immune responses, which in turn trigger systemic anti-tumor effects (20). In addition, radiotherapy induces the conversion of tumor-associated macrophages (TAM) from M2 (pro-tumor) to M1 (anti-tumor) and removes immunosuppressive cells, all of which contribute to the development of AE.

### Radiation therapy increases the expression of MHC class I molecules on the surface of tumor cells

In recent years, with the breakthrough progress in immunotherapy, immune checkpoint inhibitor (ICI) therapy has shown promising clinical responses in the treatment of various cancers. The initiation of an immune response begins with the recognition of tumor-specific antigens by major histocompatibility complex class I (MHC-I) molecules. T cells play a central role in the host immune system. Upon interaction of MHC-I and T cell receptors, the immune response is initiated with certain other additional stimuli. As a result, MHC class I-positive tumor cells are recognized and cleared by T lymphocytes or other immune cells such as macrophages (21). The loss or downregulation of MHC-I will make the cancer less visible to the immune system. Thus, MHC-I expression deficiency is associated with resistance to checkpoint blockade and overt immunotherapy (22). In addition, during immunotherapy, regression of MHC-I-high metastases and progression of MHC-I-low metastases were observed. Furthermore, defects in the IFN response pathway, which regulates MHC-I levels, have also been correlated with resistance to checkpoint immunotherapy (23). The above evidence suggests that MHC-I loss significantly reduces ICI efficacy (24–26). Radiation therapy not only increases the expression of MHC-I but also modulates the process of antigen presentation on the cell surface, enhancing the sensitivity of immune cells to tumor cells, triggering anti-tumor immunity (27, 28).

### Radiation therapy alters the tumor microenvironment

In addition to mediating DNA damage-induced cancer cell death, radiation therapy can modulate tumor immunogenicity and adjuvant properties by triggering the release of pro- and anti-inflammatory mediators, increasing immune stimulation and infiltration of immune-suppressing cells, thus transforming a “cold tumor” into a “hot tumor” and increasing tumor susceptibility to immunotherapy (29). Ionizing radiation-induced release of double-stranded DNA (dsDNA) and mitochondrial DNA (mtDNA) can activate the cGAS-STING signaling pathway and the type I IFN response, which are important for DC and T cell initiation (30, 31). Moreover, radiotherapy stimulates the release of chemokines such as CXCL9, CXCL10 and CXCL16 from tumor cells and stromal cells, which increase the infiltration of intratumoral NK, DC, and T cells, leading to enhanced anti-tumor immunity (32). However, radiation can also lead to the accumulation of immunosuppressive cell populations such as Treg and Myeloid-derived suppressor cells (MDSCs) in the tumor. The apoptotic cells that appear after RT can activate M2-type macrophages to secrete a range of anti-inflammatory cytokines such as TGF-β and IL-10, which limits the positive immunomodulatory effects of radiotherapy (33). Therefore, the effect of radiotherapy on immunotherapy is a “double-edged sword”, and it is necessary to comprehensively consider the effects of radiotherapy on cancer cells, immune cells, and various mesenchymal cells in the microenvironment, to achieve truly accurate iRT.

Metabolic reprogramming is the capacity of a cell to adjust its metabolism in response to various stimuli and stressors, allowing it to withstand external pressures and acquire new functions (34). It has been demonstrated that radiotherapy-mediated metabolic reprogramming is involved in immune regulation (35). Radiotherapy enhances the glycolysis of tumor cells (36). High glucose consumption leads to lactate production and secretion into the tumor microenvironment. Lactate exerts immunosuppressive effects by decreasing NK cell cytokinesis, increasing PD-1 expression, and activating Treg cells (37). Lactate also promotes the recruitment of MDSCs in tumor and spleen and induces “M2-like” polarization in tumor-associated macrophages (38). On the other hand, radiotherapy induces ferroptosis of tumor cells by promoting lipid peroxidation (39). Ferroptosis enhances the ability of innate immune cells to recognize tumor cells and initiates the adaptive immune response, which improves the “immune desert status” of TME. As a potential form of ICD, ferroptosis not only drives macrophage polarization toward the M1 phenotype, but also promotes intra-tumoral T cell infiltration and activation, and can even trigger a vaccine-like response, thus activating the anti-tumor immune responses (40). Taken together, radiotherapy-induced metabolic alterations play a complex and important role in immune regulation, and a better understanding of these mechanisms is essential for the development of more effective therapeutic strategies.

## Combining radiotherapy with immunotherapy in clinical practice

Based on the regulatory effects of radiotherapy on the immune system, the current radioimmunotherapy (iRT) treatment approach has been widely used in the clinical treatment of various types of cancers. Radiotherapy combined with PD-1/PD-L1 antibody drugs has achieved significant success in malignant melanoma, non-small cell lung cancer (NSCLC), head and neck squamous cell carcinoma, and some solid tumors. Additionally, radiotherapy combined with CTLA-4 inhibitors has been extensively studied and applied in melanoma, lung cancer, and other cancers (41, 42). To maximize the advantages of the iRT treatment approach in clinical settings, it is essential to thoroughly investigate the factors influencing treatment efficacy. Determining the radiotherapy dose and fractionation, selecting the irradiation site, timing of radiotherapy, and choice of immunotherapy all need to be optimized through clinical practice to identify the best combinations (43).

### Selection of radiotherapy regimens

The immune response induced by radiotherapy exhibits a “dose-dependent” nature (4), and extensive research has been conducted in clinical settings regarding the selection of radiotherapy doses and fractionation schemes. Many studies have shown that for activation of anti-tumor CD8^+^ T cell responses, a single high dose or large fractionated radiation is superior to conventional fractionated regimens (4). High-dose radiation induces enhanced double-strand breaks, leading to increased tumor cell death and release of tumor antigens and cytokines (e.g., IFN-γ), which promotes activation of cytotoxic CD8^+^ T cells (33). However, high-dose radiotherapy (≥15 Gy) might increase the proportion of Treg cells in the spleen, thereby suppressing anti-tumor immune responses (44). Therefore, the hypofractioned radiotherapy modality may be considered. A non-randomized observational study demonstrated that SBRT (48 Gy/6-8 F) induced peripheral blood T-cell activation in patients with early-stage NSCLC, mainly in increased PD-1 expression and proliferation (45). Allen et al. compared immune responses and tumor control rates with high-dose hypofractioned (8 Gy x 2) or low-dose daily fractionated (2 Gy x 10) IR combined with PD-1. The results showed that 8 Gy x 2 IR preserved peripheral and tumor-infiltrating CD8^+^ T lymphocyte activation and reduced MDSCs accumulation in comparison to 2 Gy x 10 IR (46). Furthermore, a clinical study conducted in advanced NSCLC showed that high-dose, hypofractioned SBRT (50 Gy/4 F) was superior to conventional radiotherapy (45 Gy/15 F) when combined with a PD-1 inhibitor in several efficacy endpoints (47). The above studies suggest that high-dose hypofractioned IR can preserve or enhance anti-tumor immunity and thus control primary and distant tumors. However, more efforts are needed to determine the optimal radiotherapy regimen.

In addition to the selection of radiotherapy doses and fractionation schemes, determining the irradiation site is also crucial. Lymphocyte reduction is one of the most common side effects of radiotherapy, and ensuring the integrity of the immune system is essential for effective tumor treatment. Therefore, it is important to protect lymphocytes during radiotherapy and immunotherapy to minimize damage to immune cells (48). For non-small cell lung cancer (NSCLC), in clinical practice, the primary tumor site is typically given a total dose of 50-70 Gy, while draining lymph nodes are usually irradiated with doses ranging from 45-50 Gy (49). However, since draining lymph nodes are crucial sites for T lymphocyte activation and aggregation, irradiation of tumor-free lymph nodes may inhibit the activation of anti-tumor immune responses. Furthermore, immune cells in the bone marrow and circulating in the bloodstream are highly sensitive to radiation therapy. Therefore, during radiotherapy, efforts should be made to avoid or minimize irradiation of the bone marrow and blood vessels (33).

In order to achieve the best therapeutic outcomes with radioimmunotherapy, the debate within the academic community revolves around whether these treatments should be administered synchronously or sequentially. There are numerous ongoing clinical trials exploring various combinations. For example, the PACIFIC study, which was used to evaluate the efficacy of using immunotherapy (primarily Durvalumab) after radiation therapy in patients with unresectable stage III non-small cell lung cancer (NSCLC), showed that rapid follow-up immunotherapy after radiation therapy significantly prolongs progression disease-free survival (PFS) and improves the overall survival (OS) of patients, with a manageable safety and tolerability profile (50). Another real-world study on stage III NSCLC using durvalumab consolidation after chemoradiotherapy showed a 14.3% incidence of grade 3 radiation pneumonitis in the experimental group compared to 2.5% in the observation group (51). Furthermore, results from the phase I clinical trial KEYNOTE-001 indicate that patients who received any form of radiotherapy before undergoing pembrolizumab immunotherapy experienced prolonged PFS (52). However, in patients with metastatic head and neck squamous cell carcinoma (HNSCC), simultaneous administration of nivolumab with stereotactic body radiotherapy (SBRT) did not improve objective response rates (ORR) compared to nivolumab monotherapy (53). A large number of relevant clinical trials are currently underway. For instance, A single-arm phase II trial called “Chemotherapy and Immunotherapy Induction Followed by Hypo-radiotherapy and Immunotherapy Maintenance in Locally Advanced NSCLC” (NCT05784142) was designed to determine the efficacy and safety of combining immunotherapy in association with standard chemotherapy and subsequently with hypoRT, followed by a treatment of maintenance with only immunotherapy. The investigators want to exploit the survival benefit effect of immuno-chemotherapy plus sequential hypoRT in LA-NSCLC. Another phase III clinical study “A Randomized Trial of Consolidative Immunotherapy With vs Without Thoracic Radiotherapy and/or Stereotactic Body Radiation Therapy (SBRT) After First-Line Systemic Therapy for Metastatic NSCLC” (NCT03867175) aims to compare progression-free survival of patients randomized to radiation and consolidative immunotherapy against those receiving consolidative immunotherapy alone. And to find out how effective immunotherapy and stereotactic radiotherapy are in treating patients with stage IV non-small cell lung cancer compared to immunotherapy alone after first-line systemic therapy (therapy that goes throughout the body). As a result, the optimal sequence for combining radiotherapy and immunotherapy remains undetermined, and large-scale clinical trials are still underway to investigate the optimal timing of administration of radiation and immunotherapy to improve oncologic outcomes, which may lead to new thinking in clinical care.

### Selection of immunotherapy regimens

Radiotherapy can induce anti-tumor immune responses, and a commonly studied approach is the combination of radiotherapy with immune checkpoint inhibitors (ICIs), with the most common being PD-1/PD-L1 and CTLA-4 inhibitors.

#### PD-1/PD-L1 immune checkpoint inhibitors

Under normal circumstances, the binding of PD-L1 on the surface of tissue cells with PD-1 on the surface of lymphocytes can inhibit lymphocyte function, playing a role in immune tolerance. However, many tumor cells also express PD-L1 on their surface, which can bind to PD-1 on corresponding lymphocytes, inhibiting lymphocyte function, cytokine release, and inducing lymphocyte apoptosis. This process leads to immune escape by tumor cells (54). PD-1/PD-L1 inhibitors work by disrupting these mechanisms to achieve the desired effects in cancer immunotherapy.

#### CTLA-4 immune checkpoint inhibitors

CTLA-4, expressed on the surface of activated T lymphocytes, can competitively bind to B7 molecules with CD28, inhibiting T cell activation and the secretion of interleukin IL-2. Additionally, CTLA-4 can bind to CD80/CD86 to inhibit T cell activation. These actions suppress the body’s anti-tumor immune response (55, 56). CTLA-4 inhibitors work by enhancing anti-tumor effects and boosting intra-tumoral immune responses through the mechanisms described above.

In the treatment of metastatic NSCLC, a retrospective analysis has indicated that the combination of PD-1/PD-L1 immune checkpoint inhibitors with SBRT is significantly superior to the combination of CTLA-4 immune checkpoint inhibitors with SBRT. However, this view still requires support from prospective research data (41). The CheckMate-73L study has highlighted that in the treatment of unresectable stage III NSCLC patients, the safety of the PD-1/PD-L1+CTLA-4 dual immunotherapy regimen compared to PD-1/PD-L1 inhibitor monotherapy as consolidation therapy after concurrent chemoradiotherapy was evaluated. Based on the phase II results, the dual immunotherapy group exhibited a higher incidence of grade 3 adverse events, leading to a higher discontinuation rate compared to the monotherapy group (57). In summary, compared to CTLA-4 inhibitors, PD-1/PD-L1 inhibitors in combination with radiotherapy typically have lower immune-related toxicity, are easier to administer and more highly targeted. Radiotherapy increases tumor antigen release, PD-1/PD-L1 inhibitors can deregulate T-cell suppression and synergistically enhance anti-tumor immune responses. In addition, in non-small cell lung cancer, a large number of clinical trial data support that the combination of PD-1/PD-L1 inhibitors and radiotherapy improves overall survival (OS) and progression-free survival (PFS), and significantly improves patients’ quality of life. Therefore, PD-1/PD-L1 inhibitors are generally favored for their established efficacy in NSCLC.

### Safety of radiotherapy combined with immunotherapy

The damage caused by radiotherapy is mainly attributed to the radiation site, which may result in corresponding injuries to the brain, heart, lungs, liver, and other areas. One of the most severe injuries is radiation-induced lung injury (RILI), including radiation pneumonitis and radiation pulmonary fibrosis (58). On the other hand, immunotherapy-related injuries encompass checkpoint inhibitor pneumonitis, colitis, hepatitis, immune checkpoint inhibitor-related endocrine disorders, and skin toxicity. However, it is important to draw our attention to the fact that both radiotherapy and ICIs can lead to lung inflammation, which may be an increased risk when combined, and that ICIs may trigger immune-mediated colitis manifested by diarrhea and intestinal discomfort (59). The combination of the two may lead to a wider range of inflammatory responses, requiring detection and early intervention to effectively control and minimize the occurrence of these adverse effects. Monitoring biomarkers of toxicity is essential for managing adverse effects in radiotherapy and immunotherapy, for example, by monitoring biomarkers such as circulating lymphocytes, immune-related toxicity can be recognized early and radiotherapy and immunotherapy doses can be adjusted appropriately, depending on the patient’s response and biomarker levels (60). Therefore, assessing the safety of radiotherapy combined with immunotherapy (iRT) treatment is crucial for clinical application, with radiation pneumonitis being a key concern in clinical research.

However, currently, there is no evidence indicating that the toxicity of iRT is significantly higher than the toxicity of using ICIs or RT alone (61, 62). Moreover, data from numerous clinical studies suggest that iRT typically results in grades 1 to 2 toxicity, including fatigue, skin reactions, gastrointestinal symptoms, and respiratory symptoms, which can usually be effectively managed with symptomatic treatment and supportive care. However, grade 3 (requiring medical support) or life-threatening grade 4 toxicities such as severe pneumonia or colitis are relatively rare (63). Additionally, existing clinical data indicate that iRT may be well tolerated and associated with acceptable toxicity across different types of cancer (64). Therefore, more clinical data are needed to evaluate the survival benefits and safety of iRT.

## Clinical advances of radiotherapy combined with immunotherapy in NSCLC

With the simultaneous exploration of multiple treatment modalities, the efficacy of targeted/immunotherapy in the perioperative period is gradually becoming prominent. Several clinical trials have been conducted in different stages of non-small cell lung cancer (**Table 1**).

**Table 1:** Clinical advances of radiotherapy combined with immunotherapy in NSCLC.

Study name	Study type	N	iRT	Primary outcome	Toxicity
I-SABR NCT03110978 (65)	Open-label randomized phase II trial	156	Nivolumab, SABR	4-year EFS	15%: grade 3 immunologial AEs
NCT03223155 (66)	Randomized phase I trial	37	Nivolumab, Ipilimumab, SBRT	PFS, OS	50%: pneumonitis
ASTEROID NCT03446547 (67)	Randomized multicenter open-label phase II trial	47	Durvalumab, SBRT	TTP	27%: grade 1-2 events
PACIFIC NCT02125461 (50)	Randomized, double-blind, international, phase III trial	713	Durvalumab, CRT	PFS, OS	29.9%: grade 3 or 4 AEs
LUN14-179 (68)	Single-arm, phase II, multi-institutional trial	93	Pembrolizumab, CRT	TMDD	17.2%: symptomatic pneumonitis (grade ≥2)
DETERRED NCT02525757 (69)	Phase II trial	40	Atezolizumab, CRT	Safety and tolerability	80%: grade 3 and above
DOLPHIN jRCT2080224763 (70)	Multicenter, single-arm nonrandomized controlled trial	74	Durvalumab, Radiotherapy (60 Gy)	PFS	52.9%: grade 3 or 4 AEs 5.9%: grade 5 AEs
PACIFIC-6 NCT03693300 (71)	Ongoing, multicenter, open-label, single-assignment, practice-informing, phase II trial	117	Durvalumab, CRT	The incidence of grade 3 or 4 TRAEs observed within 6 months	18.8%: grade 3 or 4 AEs
KEYNOTE-001 NCT01295827 (72)	International, multicenter, phase I trial	98	Pembrolizumab, RT	PFS, OS	45%: pulmonary toxicity
PACIFIC NCT02125461 (73)	Phase III trial	713	Durvalumab, CRT	PFS, OS	Not mentioned
SICI (74)	Single center, open-label phase I trial	15	Durvalumab, Tremelimumab, SBRT	Safety	40%: low grade irAE 14%: grade 3 irAE
PEMBRO-RT NCT02492568/MDACC NCT02444741 (75)	PEMBRO-RT: a phase II, multicenter, randomized trial MDACC: a phase I/II, single-center, randomized trial	148	Pembrolizumab, PEMBRO-RT: 24 Gy, MDACC: either 50 Gy in four fractions or 45 Gy in 15 fractions	ARR, ACR, PFS, OS	High-grade radiotherapy-related AEs were very uncommon

N, number of participants; SABR, stereotactic ablation body radiotherapy; EFS, event-free survival; AEs, adverse events; SBRT, stereotactic body radiotherapy; PFS, progression-free survival; OS, overall survival; TTP, time to progression; CRT, chemoradiation; TMDD, time to metastatic disease or death; TRAEs, treatment-related adverse events; ARR, abscopal response rate; ACR, abscopal control rate.

### iRT Treatment for early-stage NSCLC

The primary treatment for patients with early-stage NSCLC is surgical resection, but some patients still develop recurrent metastases after surgery, and many clinical trials have attempted to improve prognosis by adding adjuvant or neoadjuvant therapy. Stereotactic ablative radiotherapy (SABR) is the standard treatment for inoperable early-stage NSCLC, but regional and distant recurrent metastases are also common. Several studies have shown that immunotherapy reduces recurrence and improves patient survival after radiotherapy, and a randomized phase II trial by Joe Y Chang et al. demonstrated a significant improvement in the 4-year EFS rate and tolerable toxicity with the combination of immunotherapy and SABR (65). Another randomized phase I trial on synchronous or sequential treatment with SBRT also suggested that multimodal therapy could control metastasis and improve overall survival (66). Thus, iRT can be used in early-stage NSCLC to improve postoperative recurrence and prolong survival. The phase II clinical study ASTEROID was designed to evaluate the efficacy and safety of sequential Durvalumab treatment after SBRT in NSCLC patients with cT1~2N0M0, and the preliminary results confirmed the feasibility of SBRT in combination with Durvalumab treatment (67).

### iRT treatment for locally advanced NSCLC

Due to the insidious nature of NSCLC, a majority of patients awaiting diagnosis clinically are in the advanced stages, with locally advanced NSCLC accounting for approximately 30% of all NSCLC cases. The Durvalumab treatment arm of the PACIFIC study demonstrated significantly prolonged median overall survival (OS) and progression-free survival (PFS), with a higher 4-year PFS rate. This study established concurrent chemoradiotherapy combined with immune consolidation therapy as the standard treatment for inoperable locally advanced NSCLC (43). The LUN14-179 study (68) and the DETERRED study (69) also confirmed the effectiveness of this treatment approach. Additionally, the DOLPHIN study (70) and the PACIFIC-6 study (71) noted that Durvalumab immunotherapy combined with radical radiotherapy was synergistic and tolerable in patients with unresectable locally advanced NSCLC.

In summary, the strategy of immune consolidation therapy following concurrent chemoradiotherapy has significantly improved the progression-free survival (PFS) and overall survival (OS) of patients with inoperable locally advanced NSCLC. However, clinicians still need to be highly aware of the adverse effects associated with this mode of treatment.

### iRT treatment for advanced NSCLC

In the treatment of advanced NSCLC, the focus of clinical research is on the combination of SBRT with immune consolidation therapy. In a secondary analysis of KEYNOTE-001, it was found that patients with advanced NSCLC who had received any area or extracranial radiotherapy in addition to Pembrolizumab treatment showed significant improvements in overall survival (OS) and progression-free survival (PFS) compared to those who had only received Pembrolizumab (72). In addition to this, in a phase III placebo-controlled PACIFIC trial, researchers found that consolidation of durvalumab treatment after radiotherapy significantly improved PFS and OS with a manageable safety profile (73). The SICI study also indicated that in patients with advanced NSCLC, our combination of durvalumab, tremelimumab and SBRT use is safe and feasible for primary tumors (74).

With the advancement of radiotherapy technology, the application of Stereotactic Body Radiation Therapy (SBRT) in advanced NSCLC is becoming increasingly widespread. Theelen conducted a combined analysis of data from the PEMBRO-RT and MDACC clinical studies involving the treatment of advanced NSCLC patients with radiotherapy combined with Pembrolizumab. The results demonstrated that SBRT combined with Immune Checkpoint Inhibitors (ICIs) significantly improved the prognosis of patients with advanced NSCLC (75).

## Conclusion and future perspectives

According to current clinical data, the combination of radiotherapy and immunotherapy is considered to be an effective treatment for NSCLC. Several studies have shown that iRT can significantly prolong the overall survival (OS) and progression-free survival (PFS) of patients with advanced NSCLC with an acceptable safety profile, and not only improve local tumor control, but also induce systemic immune response and control distant metastasis through the “abscopal effect”. However, the most effective combination and sequence of iRT in non-small cell lung cancer (NSCLC) requires comprehensive clinical trial data to guide clinical protocols.

iRT is regarded as a potential breakthrough in the field of tumor therapy, which can improve the therapeutic effect and reduce the therapeutic resistance. In the direction of future development, on the one hand, biomarkers predicting the combined application of radiation therapy and immunotherapy can be investigated, which may involve factors such as the immune characteristics of the tumor microenvironment and the genetic variation of tumor cells (76). On the other hand, the application of iRT in other solid tumors can be explored, including but not limited to lung cancer, melanoma, pancreatic cancer. Through clinical trials and studies, the efficacy of combination therapy in different cancer types can be verified and the best treatment options can be explored (Reardon, 2024)^1^. There is also a need to explore new therapeutic strategies, such as enhancing the effect of immunotherapy through gene editing technology, or using nanotechnology to improve the targeting of radiation therapy (Liu et al., 2024)^2^. The research and practice of the above development directions can further promote the development of the combined application of radiation therapy and immunotherapy, provide more effective treatment options for tumor patients, and bring new breakthroughs in the field of solid tumor treatment.

## Footnotes

1 Reardon S. (2024). First cell therapy for solid tumours heads to the clinic: what it means for cancer treatment. *Nature*. (Ahead of print). doi: 10.1038/ d41586-024-00673-w

2 Liu H, Guo Z, Wang P. (2024). Genetic expression in cancer research: Challenges and complexity. *Gene Rep*. (Ahead of print). doi: 10.1016/j.genrep.2024.102042
